# Why Young People With Eating Disorder Symptoms Do Not Seek Help—Exploring Barriers to Help‐Seeking

**DOI:** 10.1002/eat.24515

**Published:** 2025-08-12

**Authors:** Johanna Stadler, Markus Moessner, Katja Becker, Alisa Hiery, Silke Diestelkamp, Heike Eschenbeck, Vera Gillé, Michael Kaess, Julian Koenig, Christine Rummel‐Kluge, Elisabeth Kohls, Rainer Thomasius, Stephanie Bauer, the ProHEAD Consortium, Michael Kaess, Stephanie Bauer, Rainer Thomasius, Christine Rummel‐Kluge, Heike Eschenbeck, Hans‐Joachim Salize, Katja Becker, Sabrina Bonnet, Johannes Feldhege, Christina Gallinat, Stella Hammon, Julian Koenig, Sophia Lustig, Markus Moessner, Fikret Özer, Regina Richter, Franz Resch, Johanna Stadler, Steffen Luntz, Silke Diestelkamp, Anna‐Lena Schulz, Sabrina Baldofski, Sarah‐Lena Klemm, Elisabeth Kohls, Sophia Müller, Lina‐Jolien Peter, Mandy Rogalla, Vera Gillé, Johanna Jade, Laya Lehner, Elke Voss, Alisa Hiery, Jennifer Krämer

**Affiliations:** ^1^ Heidelberg University, Institute of Psychology Heidelberg Germany; ^2^ Center for Psychotherapy Research University Hospital Heidelberg Heidelberg Germany; ^3^ Department of Child and Adolescent Psychiatry, Psychosomatics and Psychotherapy Philipps‐University of Marburg Marburg Germany; ^4^ University Hospital Hamburg‐Eppendorf, German Center for Addiction Research in Childhood and Adolescence Hamburg Germany; ^5^ Department of Educational Psychology and Health Psychology University of Education Schwäbisch Gmünd Schwäbisch Gmünd Germany; ^6^ Clinic of Child and Adolescent Psychiatry, Center for Psychosocial Medicine University Hospital Heidelberg Heidelberg Germany; ^7^ University Hospital of Child and Adolescent Psychiatry and Psychotherapy University of Bern Bern Switzerland; ^8^ Department of Child and Adolescent Psychiatry, Psychosomatics and Psychotherapy Faculty of Medicine and University Hospital Cologne, University of Cologne Cologne Germany; ^9^ Department of Psychiatry and Psychotherapy Medical Faculty, University of Leipzig Leipzig Germany; ^10^ German Center for Addiction Research in Childhood and Adolescence University Medical Center Hamburg‐Eppendorf Hamburg Germany; ^11^ German Center for Mental Health (DZPG), Partner Site Mannheim/Heidelberg/Ulm Heidelberg Germany

**Keywords:** adolescents, Classification and Regression Trees, decision tree, eating disorders, help‐seeking, youth

## Abstract

**Objective:**

Rates of help‐seeking and treatment uptake are low in eating disorders. Delayed initiation of treatment has a negative impact on prognosis and treatment outcome and leads to a higher burden on the healthcare system. The aim of this study was to explore factors associated with help‐seeking and their interactions in a large sample of adolescents and young adults with current symptoms of an eating disorder.

**Methods:**

Based on the Classification and Regression Trees (CART) algorithm, the data collected within the German school‐based project ProHEAD (*N* = 9796; age: 12–25 years) were used to estimate a decision tree to classify students into help‐seekers and non‐help‐seekers for a mental health issue.

**Results:**

Of those screened, 13% reported substantial current eating disorder symptoms (*N* = 1273). Out of those, 77.3% reported that they did not seek formal help (i.e., from a mental health professional). The absence of suicidal ideation and emotional problems, as well as a low level of education and openness to mental health issues, was characteristic of those who did not seek help for a mental health problem. Emotional problems, suicidality, and depressive symptoms were identified as the most important factors associated with general help‐seeking.

**Discussion:**

In line with previous research, our findings indicate that individuals with eating disorder symptoms are more likely to seek help when other mental health issues are present. Public health efforts should aim to promote awareness and increase knowledge of eating disorders.

**Summary**

- Most individuals affected by an eating disorder do not seek help. If left untreated, this contributes to prolonged suffering, a poorer long‐term course of illness, and high healthcare costs.
- This study is the first to explore how factors predicting help‐seeking interact in youth, using the Classification and Regression Trees (CART) algorithm.
- Findings suggest that mental health knowledge and awareness of mental health problems beyond eating disorders should be considered in prevention measures.

## 1 Introduction

In most cases, eating disorders first occur in adolescence or early adulthood and have serious consequences for health, quality of life, and well‐being (Solmi et al. 2022). Anorexia nervosa, for example, has one of the highest mortality rates among mental disorders; suicidal thoughts and suicide attempts are not rare in eating disorders (Chesney et al. 2014; Rania et al. 2021). Even emerging symptoms or subthreshold forms of eating disorders cause substantial impairment (Fabry et al. 2024). Despite that, only a minority of affected individuals seek and receive treatment (Ali et al. 2025; Fitzsimmons‐Craft et al. 2019; Lipson et al. 2017). Estimates of those receiving eating disorder treatment range from 13.6% (Sonneville and Lipson 2018) to 23.3% (Ali et al. 2025; Hart et al. 2011). Among adolescents affected by an eating disorder, around 10% reported seeking help for a body image problem (Fatt et al. 2020). For adolescents and adults who do seek help, it takes on average 21.3 months until they receive specialized treatment (Austin et al. 2021).

Delayed help‐seeking and low treatment uptake are not only associated with considerable negative consequences such as worse treatment outcomes, poorer long‐term prognosis, and higher health care costs, but also with prolonged suffering, lower social participation, and dropping out of school (Ambwani et al. 2020; Austin et al. 2021; Fernandez‐Aranda et al. 2021). Hence, there is a clear need to reduce the time between eating disorder onset and adequate professional treatment.

### 1.1 Barriers and Facilitators of Seeking Help

Various factors contribute to the treatment delays in eating disorders. Barriers in the healthcare system include availability, accessibility, and costs of eating disorder treatment (Allen et al. 2023; Radunz et al. 2023). Eating disorders often go undetected or are treated too late, mainly due to a lack of knowledge among healthcare professionals or the pursuit of a “watch and wait” strategy.

However, the main delay in seeking specialized treatment occurs *before* help is sought, primarily due to patient‐related factors, such as illness denial (Austin et al. 2021; Beat 2017; Volpe et al. 2019). As eating disorders are one of the most stigmatized mental disorders (Ebneter et al. 2011; Roehrig and McLean 2010), stigma and shame are also the most frequently reported barriers to help‐seeking (Ali et al. 2017). Furthermore, the inability to perceive the severity of the illness as well as ambivalence to change are common and may also reduce the likelihood of seeking professional help (Ali et al. 2017; Daugelat et al. 2023; Innes et al. 2017; Nicula et al. 2022; Radunz et al. 2023; Regan et al. 2017). Barriers like these apply in particular to young people. Similarly, in terms of demographic factors, male gender, belonging to an ethnic minority, and a lower educational level have been found to negatively impact help‐seeking (Regan et al. 2017; Thapliyal et al. 2020).

A desire for recovery and social support from others seem to facilitate help‐seeking (Ali et al. 2017; Daugelat et al. 2023; Nicula et al. 2022; Regan et al. 2017). In addition, greater subjective severity of the eating disorder and related distress also leads people affected to approach sources of help (Ali et al. 2017; Nicula et al. 2022). Often, affected individuals seek help for issues like weight loss, psychological impairment, mental or physical health problems associated with the eating disorder, rather than the eating disorder itself (Hart et al. 2011; Ivancic et al. 2021; Striegel Weissman and Rosselli 2017).

### 1.2 The Present Study

Previous research has almost exclusively examined single factors associated with help‐seeking for an eating disorder, thus neglecting possible interactions between these factors. The present study, therefore, explored the relevance of specific predictors and their interaction in relation to help‐seeking in a large sample of adolescents and young adults with eating disorder symptoms. This exploratory study addresses two objectives related to why young people refrain from seeking help:

1. Which factors predict a lack of help‐seeking among youth with eating disorder symptoms?
2. What are the specific interactions of the factors predicting help‐seeking?

To answer these questions, we applied the Classification and Regression Trees (CART; Breiman et al. 1984) algorithm, which allowed us to flexibly explore a range of heterogeneous predictor variables and shed light on their interactions. Applying CART results in a robust, comprehensive, and intuitive decision tree that only features a fraction of the available data, making it easy to draw practical implications, as it clearly indicates the importance of each predictor for help‐seeking in youth with eating disorder symptoms.

## 2 Methods

### 2.1 Participants and Procedures

Data were collected as part of the screening assessment of the German multicenter ProHEAD project (Promoting Help‐seeking using E‐technology for Adolescents; Kaess and Bauer 2019). The ProHEAD project aimed to encourage participants with mental health problems to seek help, to prevent disorders in those with subclinical symptoms, and to improve the mental health of unaffected participants. Recruitment took place in randomly selected schools in five regions across Germany. Inclusion criteria were proficiency in German, age of 12 years or older, Internet access, and informed consent from both the student and parents. Participants were assigned to five subprojects based on their mental health status indicated by the screening assessment and invited to participate in one of the interventions. The screening assessments were conducted using the ASMO software (Wilhelm et al. 2020) between November 2018 and February 2022. The assessments were conducted at the participating schools until the outbreak of the COVID‐19 pandemic. After the onset of the pandemic, students were still recruited via their schools, but could complete the assessments from home. Ethical approval was granted by the Medical Faculty at Heidelberg University (Study‐ID: S‐086/2018) and the respective institutional review boards of all involved study sites (Hamburg, Schwäbisch Gmünd, Leipzig, Marburg).

For the purpose of this study, substantial eating disorder symptoms were defined as a score of ≥ 2.3 in the *Child Eating Disorder Examination Questionnaire* (ChEDE‐Q; Fairburn and Beglin 2008; TODAY Study Group 2007), as determined by Mond et al. (2004) and in accordance with previous studies (e.g., Brockmeyer et al. 2013; Ruwaard et al. 2013). Of all *N* = 9796 participants who completed the ProHEAD screening assessment, *n* = 9509 (97.1%) answered both the *Actual Help‐seeking Questionnaire* (AHSQ; Rickwood and Braithwaite 1994) (outcome) and the ChEDE‐Q, and all predictors analyzed in this study. Of these, *n* = 1273 (13.0%) exceeded the ChEDE‐Q cutoff score and were included in the present study. The CART algorithm is robust regarding incomplete data sets. In rare cases of missing data, a variable with the best similar pattern in relation to the outcome is used by the CART algorithm.

### 2.2 Measures

The 22‐item *ChEDE‐Q* global score, which assesses the severity of psychopathology related to eating disorders in the past 28 days in children, was used to select the study sample. Cronbach's alpha indicated high reliability in this study (*α* = 0.80).

The primary outcome, that is, utilization of professional help for a mental health issue, was measured by the AHSQ. The AHSQ measures actual general help‐seeking by asking whether participants have sought help from specific formal or informal sources in the past 12 months or prior. For the present study, participants who reported seeking help from a formal source (i.e., school psychologist, child and adolescent psychotherapist or psychiatrist, counselor, general practitioner, and teacher or school counselor) were assigned to the “help‐seeking” class, irrespective of when they sought help. Those who did not seek help or did so only from informal sources (e.g., parents) were assigned to the “no help‐seeking” group. Categorization of formal general help‐seeking was based on definitions by Rickwood and Braithwaite (1994) and Rickwood et al. (2005).

Twenty‐one potential predictors were explored in the analysis, including items on age, sex, and potential migration background of parents or the participant. Socioeconomic status was measured by the four‐item *Family Affluence Scale* (FAS; Currie et al. 2008). Participants also reported their school type. In Germany, after 4 years of elementary school, students are assigned to different types of secondary schools: “Haupt‐ und Werkrealschule” (secondary general school, Grades 5–9/10), “Realschule” (secondary school, Grades 5–10), “Gymnasium” (academic secondary school culminating in an exam granting access to university, Grades 5–12/13), and “Gemeinschaftsschulen/Oberschule/Stadtteilschule” (comprehensive secondary school, Grades 5–9/10). “Berufsschule” follows on from secondary schools and combines theoretical and practical on‐the‐job training.

The 25‐item *Strengths and Difficulties Questionnaire* (SDQ; Goodman 1997) measures behavioral and emotional problems in 11‐ to 17‐year‐olds in the past 6 months. The SDQ consists of five subscales with scores ranging from 0 to 10: Emotional Problems, Conduct Problems, Peer Problems, Hyperactivity, and Prosocial Behavior. The first four subscales are added up to a total score ranging from 0 to 40; higher scores indicate high general psychopathological distress. In this study, Cronbach's alpha indicated acceptable reliability (*α* = 0.71). The nine‐item *Patient Health Questionnaire* (PHQ‐9; Kroenke et al. 2001) was used to assess depressive symptoms in the past 2 weeks. The total score ranges from 0 to 27. Values above 10 indicate moderate, above 15 moderately severe, and above 20 severe depressive symptoms. In this study, Cronbach's alpha indicated high reliability (*α* = 0.83). The five‐item *Paykel Suicide Scale* (PSS; Paykel et al. 1974) evaluates suicidal behavior in the past 12 months on a dichotomous scale with scores ranging from 0 to 5. Higher scores indicate a high severity of suicidal ideation. In this study, Cronbach's alpha indicated high reliability (*α* = 0.80). The 10‐item *Alcohol Use Disorders Identification Test* (AUDIT; Babor et al. 2001) measures alcohol intake, potential dependence on alcohol, and harm experienced due to alcohol consumption in the past 12 months. The total score ranges from 0 to 40. Values above 8 suggest hazardous alcohol consumption, and values above 15 indicate a moderate to severe alcohol use disorder. In this study, Cronbach's alpha indicated acceptable reliability (*α* = 0.77). The six‐item *CRAFFT‐d* (Tossmann et al. 2009) assesses lifetime alcohol use and alcohol‐related risk as well as alcohol use disorder in 12‐ to 21‐year‐olds. The total score ranges from 0 to 5, with scores above 2 indicating potential alcohol‐related problems. In this study, Cronbach's alpha indicated a medium reliability (*α* = 0.66).

Current key eating disorder symptoms, such as frequency of measures to counteract weight gain, as well as body weight and height, were measured using the *Short Evaluation of Eating Disorders* (SEED; Bauer et al. 2005). Age and gender specific percentiles of the *BMI* were calculated and assigned to the corresponding categories for ease of interpretation: extreme underweight (< 5th percentile), underweight (< 10th percentile), healthy weight, overweight (> 85th percentile), and extreme overweight (> 95th percentile). The five‐item *Weight Concerns Scale* (WCS; Killen et al. 1994) measures current concern regarding body weight and shape. The WCS score ranges from 0 to 100, with higher values indicating high concern. In this study, Cronbach's alpha indicated a medium reliability (*α* = 0.63).

The two subscales of the 24‐item *Inventory of Attitudes toward Seeking Mental Health Services* (IASMHS; Mackenzie et al. 2006) on current Psychological Openness and Indifference to Stigma were used, with scores ranging from 0 to 32. The subscale Help‐seeking Propensity was omitted due to its conceptual overlap with the outcome. In this study, Cronbach's alpha indicated acceptable reliability (*α* = 0.77). Familiarity with a person affected by a mental illness was assessed with one item. A 25‐item self‐report questionnaire (*Fragebogen zur Sozialen Distanz* [Questionnaire on Social Distance]; Schulze et al. 2003) was used to assess current social distance concerning persons affected by a mental health problem (e.g., “Someone mentally ill should not attend a normal school”), negative stereotypes (e.g., “If someone becomes mentally ill, it is usually their own fault”), and perceived stigmatization (e.g., “Most adults have prejudices against mentally ill people”). Cronbach's alpha indicated acceptable reliability in this study (*α* = 0.73).

### 2.3 Statistical Analyses

Statistical analyses were conducted using R (R Core Team 2022) and SPSS version 29 (IBM Corp 2022).

In order to explore predictors of help‐seeking, the CART algorithm (Breiman et al. 1984) was applied. CART is particularly well‐suited for the exploratory analysis of complex associations in data. It is a powerful algorithm to explore higher‐order interactions of predictor variables. The algorithm aims to find values of the included predictor variables that adequately split the data into subgroups of participants who either (1) did not seek professional help or (2) sought professional help.

The tree starts with a single node that branches out into further internal nodes; nodes that do not split further are called leaf nodes. The CART algorithm uses the Gini impurity index to iteratively split data into homogeneous subgroups regarding the outcome (help‐seeking), selecting the variable and split point that maximally reduces impurity. A predicted class is assigned to each subgroup by majority vote (i.e., Class 1: *no help‐seeking*, Class 2: *help‐seeking*). The recursive splitting process proceeds until all leaf nodes are pure, containing only cases from a single class. However, such trees tend to overfit and lack generalizability. Overfitting can be managed either by creating an overfitted tree that is then pruned (cost‐complexity pruning) or by applying pre‐specified stopping criteria, such as minimum node size and maximum tree depth (Berk 2009, 2020; Hayes et al. 2015; Strobl et al. 2009). In this study, the latter “pre‐pruning” method was used, as a sufficiently large sample size per node is required to make meaningful assumptions for future prevention efforts. A maximum tree depth (i.e., maximum number of nodes from root to the furthest node) of 5 nodes with a minimum number of 100 observations in nodes and 50 observations in leaf nodes was predefined.

Due to the imbalanced distribution of the outcome, the relative penalty associated with incorrect classification (misclassification costs) was adapted accordingly, that is, the mistake of wrongly assigning a case to the class of *no help‐seeking* is three times as costly. A 10‐fold cross‐validation was conducted. The predictor that produces the greatest reduction in Gini impurity index, yielding a more homogeneous tree, is defined as the most important. Relative predictor importance is standardized as the percentage of improvement in relation to the most important predictor, whose importance is 100%. Highly important variables contribute substantially to the model's overall predictions and tend to appear higher up in the tree. However, this is not always the case: a variable of high relative importance may be omitted if it is strongly correlated with another predictor, rendering it redundant for the prediction of the outcome. Moreover, at each node, the CART algorithm selects the split that improves homogeneity the most, which may favor the use of a less important variable. Ultimately, the tree reflects both interactions among variables and the optimization of homogeneity by the CART algorithm.

## 3 Results

### 3.1 Sample Characteristics

A total of 1273 adolescents and young adults from Germany who completed the outcome questionnaire and reported substantial eating disorder symptoms were included in the analysis (13.0% of the full sample). Most participants were female (84.2%), and their age ranged from 12 to 25 years (*M* = 15.39, SD = 2.26). Among all participants, 44.0% attended the “Gymnasium,” 12.9% the “Realschule,” 8.9% “Haupt‐ und Werkrealschule,” and 19.6% the “Gemeinschaftsschule/Oberschule/Stadtteilschule.” “Berufsschule” was attended by 10.9% of participants. Distribution of school types differed marginally from the total sample. Most participants had not sought help from a formal source (77.3%). Of those seeking help, most of them turned to child and adolescent psychotherapists (54.3%) and/or psychiatrists (36.0%), followed by their teacher or school counselor (24.6%). For more detailed sample characteristics, see Table S1.

### 3.2 CART

**FIGURE 1:**
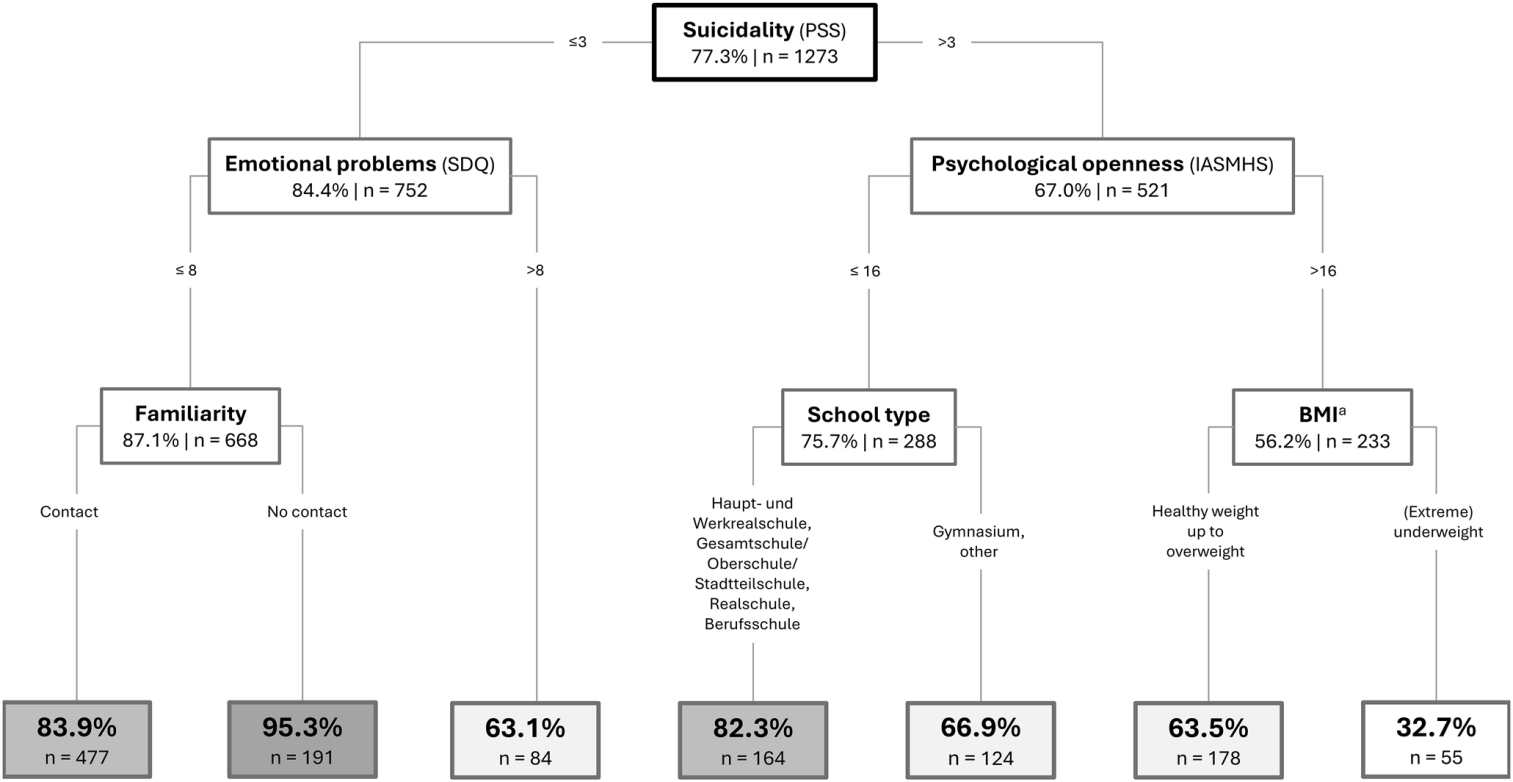
Decision tree model for the prediction of help‐seeking using CART. *Note*. Boxes represent nodes displaying the number of participants assigned to that node and the percentage of participants classified as “no help‐seeking.” ^a^BMI percentiles were categorized as follows: Extreme underweight (< 5th percentile), underweight (< 10th percentile), healthy weight, overweight (> 85th percentile), and extreme overweight (> 95th percentile).

The CART model comprised a total of 13 nodes, of which 7 were leaf nodes, with a tree depth of 3 (Figure 1). Each node shows the number of people assigned to that node and the percentage of participants at that node who have *not* sought help (e.g., 77.3% of no help‐seeking at the root node). The accuracy of classifications was 70%, that is, of 1273 observations, 891 were classified correctly (Table 1).

**TABLE 1:** Classification accuracy for the decision tree model.

Observed	Predicted	
	No help‐seeking	Help‐seeking
No help‐seeking	717 (**72.9%**)	267 (27.1%)
Help‐seeking	115 (39.8%)	174 (**60.2%**)

*Note*: Highlighted percentages indicate the proportion of correctly assigned cases.

**FIGURE 2:**
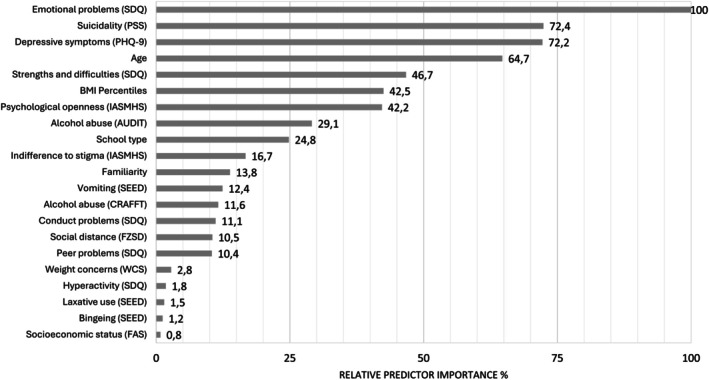
Relative importance of included predictor variables.

Of the 21 predictors included in the model, six predictors were selected for generating the decision tree: suicidality (PSS), “Emotional Problems” (SDQ), “Psychological Openness” (IASMHS), familiarity, school type, and BMI percentiles. Figure 2 shows the relative predictor importance. The SDQ scale “Emotional Problems” emerged as the most important predictor, followed by suicidality and depressive symptoms. Binge eating, the use of laxatives, and “Hyperactivity” (SDQ) showed limited importance.

In the CART model, the first split was based on suicidality. Within the subgroup of individuals with low suicidality scores (≤ 3, *n* = 752), 36.9% of the individuals with greater “Emotional Problems” (> 8) sought help, whereas the vast majority of individuals with scores ≤ 8 did not seek help (87.1%). In the subgroup of individuals with elevated suicidality scores (> 3, *n* = 521), two‐thirds did not seek professional help (67%). Within this subgroup, those with higher scores of “Psychological Openness” (> 16) were more likely to seek help.

Overall, the highest probability of help‐seeking was found for (extremely) underweight participants who reported increased suicidality and “Psychological Openness.” Notably, these participants represented the smallest subgroup in the tree (*n* = 55). Apart from the leaf node sizes, observations were distributed relatively balanced after each split between the resulting nodes. Among the leaf nodes, the largest group (*n* = 477) comprised participants with suicidality scores ≤ 3, “Emotional Problems” ≤ 8, and prior contact with a person affected by a mental illness. Finally, despite substantial eating disorder‐related impairment, fewer than 5% of individuals who reported low suicidality, no severe “Emotional problems,” and no familiarity with someone affected sought help.

## 4 Discussion

The present study explored predictors of help‐seeking for a mental health problem in adolescents and young adults with substantial eating disorder symptoms. To our knowledge, this is the first study to use the CART algorithm to explore the complex association between a variety of factors that may influence general help‐seeking.

Consistent with prevalence estimates of (subthreshold) eating disorders reported in previous research (e.g., Hilbert et al. 2012), 13% of all screened participants exhibited current eating disorder symptoms. Despite reporting marked eating disorder psychopathology, the majority did not seek help for a mental health problem from a formal source (77.3%), consistent with low rates of help‐seeking in prior research (e.g., Ali et al. 2025).

Starting from the top of the tree, elevated suicidal tendencies were the strongest predictor for help‐seeking (33%). Among participants with low suicidality, 84.4% did not seek help. In this subgroup, help‐seeking increased when additional emotional problems were reported (36.9%). Participants with low suicidality and low emotional problems rarely sought help (12.9%). Within this subgroup, being familiar with someone affected by a mental health problem facilitated help‐seeking. Strikingly, the combination of low suicidality, a low level of emotional problems, and *not* being familiar with someone affected meant that almost no help was sought (95.3%). This was the lowest rate in the model; 15% of the sample (*n* = 191) fell into this category. Familiarity with someone affected is the strongest predictor of help‐seeking for adolescents who primarily reported eating disorder symptoms. This finding underscores the value of prevention efforts that involve or are partially delivered by individuals with lived experience of mental illness (Griffiths et al. 2014; Thomas et al. 2015).

Roughly one‐third of the participants with elevated suicidality in addition to their eating disorder symptoms sought help. Within this subgroup, psychological openness was the strongest predictor of help‐seeking: 24.3% of those with low openness sought help, compared to 43.8% with high openness. Psychological openness refers to the extent to which someone is willing to acknowledge the presence of a psychological problem and to the prospect of seeking help. Being psychologically open was associated with greater experience and knowledge in the mental health field (Shahwan et al. 2020). For individuals with substantial eating disorder symptoms who exhibit suicidality, psychological openness is crucial when seeking help.

BMI proved to be an important predictor only within the subgroup of participants with increased suicidality and psychological openness: (extreme) underweight (< 10th percentile) led 67.3% to seek help, the highest rate among all nodes. This aligns with findings on elevated help‐seeking in cases of anorexia nervosa compared to other eating disorders (Bartholdy et al. 2017; Ciao et al. 2022). This may be attributed to the behavioral and physical consequences of anorexia nervosa, which are more readily recognized by others. Although BMI appeared to be highly predictive of help‐seeking for participants with elevated suicidality and psychological openness, this subgroup, however, represents only 18% of the sample. Hence, for most participants with eating disorder symptoms, BMI was not a predictor of help‐seeking.

Among participants with elevated suicidality and *low* psychological openness, the educational level played a critical role: higher help‐seeking rates for schools with higher educational levels (33.1%) compared to the other school types (17.7%). A reason for this could be that people with a lower educational level tend to use maladaptive coping strategies in crises, such as self‐blame (Molero Jurado et al. 2021), which may hinder their search for help. In addition, people with higher education show greater mental health literacy (Jorm et al. 1997), which in turn makes it easier for them to seek help. Beyond involving people with lived experience of mental illness, schools with lower educational levels may represent a particularly promising target for preventive interventions.

It is often the presence of *other* mental health problems that prompts people with eating disorders to seek professional help (Ivancic et al. 2021). In this case, suicidality and emotional problems increased the likelihood of help‐seeking. In contrast, only 12.9% of the participants with low levels of either of these additional issues sought help, despite reporting substantial eating disorder symptoms. The relative importance of predictors (Figure 2) provides further proof that additional psychological problems are the most important influence on help‐seeking in individuals with symptoms of an eating disorder. Individuals affected by eating disorders often fail to recognize the severity of their symptoms and deny having eating disorder‐related mental health problems (Lipson et al. 2017). Denial may become untenable if additional symptoms such as suicidal thoughts or emotional problems are perceived as highly distressing and burdensome. Co‐occurrence of other mental health problems is common in eating disorders and leads to a more severe course of the illness (Rania et al. 2021; Vall and Wade 2015). The resulting decline in well‐being may fuel the search for help, as supported by this exploratory study's finding that additional mental health issues substantially increased the likelihood of seeking help.

Although stigma has been frequently reported as a barrier to help‐seeking, in our study, indifference to stigma was considerably less influential compared to other mental health issues (Figure 2). This aligns with a recent review, which suggested that only denial of the eating disorder's severity and perceived inability of others to provide help predict help‐seeking behavior (Radunz et al. 2023).

Our results complement existing literature, which primarily focused on main effects or two‐way interactions of help‐seeking predictors. By exploring the higher‐order interactions, CART enables the identification of distinct subgroups with eating disorder symptoms and the specific predictors relevant to each subgroup. For example, the predictive value of BMI, educational level, and familiarity only appears in specific subgroups (three‐way interactions). This approach broadens our understanding of help‐seeking and supports the development of tailored interventions for distinct target populations.

### 4.1 Strengths and Limitations

Various single factors hindering help‐seeking for an eating disorder have been identified. Nevertheless, the help‐seeking rate has remained alarmingly low, leading to a prolonged duration of untreated illness and negative effects on the individual and healthcare level. To date, research on help‐seeking for eating disorders has mainly focused on adult samples. To our knowledge, this is the first study to explore help‐seeking in a large sample of 12‐ to 25‐year‐olds with eating disorder symptoms. This study advances the field by using the CART algorithm to explore interactions between several factors that impede or facilitate help‐seeking. This study aimed at a deeper understanding of the help‐seeking process, which may ultimately result in new approaches to reduce the treatment gap (Ali et al. 2025). The resulting decision tree can serve as a starting point for future research.

Due to the exploratory nature of our study, results must be interpreted cautiously. Given the model's limited classification accuracy, a lot remains unknown about why participants did or did not seek help. The model may lack predictors that further improve classification. One of the main limitations lies in the difficulty of establishing the chronological order of events, especially regarding the onset of eating disorder symptoms and when help was sought. The ChEDE‐Q captures eating disorder symptoms over the past 28 days. In contrast, the AHSQ captures help‐seeking for a mental health problem in the past and in general (not specifically for eating disorders). Therefore, the main reason for seeking help remains unclear, and, more importantly, help‐seeking may have occurred *before* the onset of eating disorder symptoms. Like other cross‐sectional studies, our study cannot mirror specific longitudinal developments. In addition, this study relies entirely on self‐report data, and participants who denied their symptoms in the EDE‐Q would have been excluded. For male participants in particular, gender‐specific inclusion criteria might be more appropriate. Furthermore, external cross‐validation would have strengthened the approach. While our study provides valuable insights into whether affected individuals seek help, there are several questions warranting further exploration given the limitations discussed.

### 4.2 Further Directions

Given the results of this study, attention should be paid to two aspects regarding the low help‐seeking rate in eating disorders: one, the presence of mental health problems beyond eating disorder‐related impairment, and two, mental health literacy. This study underscores the importance of being familiar with and open to mental health issues and having a higher educational level as facilitators of help‐seeking. In particular, the association between additional symptoms (e.g., suicidality), psychological openness, and severe eating disorder symptoms (e.g., [extreme] underweight) was highlighted by our findings. This presents opportunities for school‐based prevention approaches typically applied in this age group. To date, several evidence‐based programs aim to raise awareness and prevent general or specific mental health problems. However, a recent systematic review shows that the specific conditions for effective implementation of such programs in individuals at risk for or affected by eating disorders remain unclear (McClure et al. 2024). Based on our findings, we recommend complementing existing programs with components that improve knowledge, familiarity, and openness about mental illness and health, so that they not only aim at counteracting the development of eating disorders but also at facilitating help‐seeking in those who are affected. Likewise, synergy effects from other prevention programs, e.g., against suicidal tendencies or depression, could be beneficial.

### 4.3 Conclusion

Low help‐seeking is a major challenge in eating disorders. Improving help‐seeking requires a better understanding of contributing factors and their interplay. The results of the present study underline the need for public health and prevention efforts, that is, sound sources of knowledge that educate, promote self‐recognition, and increase openness toward (one's own) mental health problems among adolescents and young adults. This study points to the value of the exploratory CART method in deepening our understanding of the factors related to help‐seeking for an eating disorder and can serve as a foundation for future research and intervention development.

## Author Contributions

**Johanna Stadler:** conceptualization, data curation, formal analysis, methodology, validation, visualization, writing – original draft, writing – review and editing. **Markus Moessner:** conceptualization, methodology, data curation, validation, writing – review and editing. **Katja Becker:** writing – review and editing, funding acquisition. **Alisa Hiery:** writing – review and editing. **Silke Diestelkamp:** writing – review and editing. **Heike Eschenbeck:** funding acquisition, writing – review and editing. **Vera Gillé:** writing – review and editing. **Michael Kaess:** funding acquisition, project administration, supervision, writing – review and editing. **Julian Koenig:** writing – review and editing. **Christine Rummel‐Kluge:** funding acquisition, writing – review and editing. **Elisabeth Kohls:** writing – review and editing. **Rainer Thomasius:** writing – review and editing, funding acquisition. **Stephanie Bauer:** conceptualization, funding acquisition, project administration, supervision, validation, writing – review and editing.

## Ethics Statement

Ethical approval for the ProHEAD project was granted by the Medical Faculty at the University of Heidelberg (Study: S‐086/2018) and all involved study sites.

## Consent

Students (and parents, if applicable) provided informed consent.

## Conflicts of Interest

The authors declare no conflicts of interest.

## Supporting information


**Table S1:** Sample characteristics (*N* = 1273).

## Data Availability

Research data are not shared.
