# Supplementary material for: Why Young People With Eating Disorder Symptoms Do Not Seek Help—Exploring Barriers to Help‐Seeking
Source: Int J Eat Disord. 2025 Aug 12;58(11):2147–56. doi: 10.1002/eat.24515 (PMC12605834; doi:10.1002/eat.24515)
Supplement: Supplementary file 1 — Table S1: Sample characteristics (N = 1273). [file EAT-58-2147-s001.pdf]

Supporting Information

Table 1

Sample characteristics (N=1,273)

Measure		*n (%)*	*M (SD)*	Range
Age			15.39 (2.26)	12-25
Sex	Female	1072 (84.21)		
	Male	201 (15.79)		
Eating disorder psychopathology (ChEDE-Q)	Global score		3.4 (.85)	2.3-5.95[a]
Help-seeking (AHSQ)	Help-seeking	289 (22.70)		
	No help-seeking	984 (77.29)		
Socioeconomic status (FAS)			6.16 (1.91)	0-9
School type	Gymnasium	560 (43.99)		
	Realschule	164 (12.88)		
	Hauptschule	113 (8.88)		
	Gesamtschule	249 (19.56)		
	Berufsschule	139 (10.92)		
	Other school type	43 (3.38)		
	Missing	5 (0.00)		
Strengths and difficulties (SDQ)	Global score		16.53 (5.07)	0-34
	Emotional problems		6.22 (2.42)	0-10
	Conduct problems		2.41 (1.67)	0-9
	Peer problems		3.58 (1.85)	0-10
	Hyperactivity		4.33 (2.12)	0-10
Depressive symptoms (PHQ-9)			13.36 (5.78)	0-27

Cont.

Measure		*n (%)*	*M (SD)*	Range
Suicidality (PSS)			2.66 (1.63)	0-5
Alcohol abuse (AUDIT)			3.69 (5.31)	0-31
Alcohol abuse (CRAFFT-d)			1.13 (1.4)	0-6
Key eating disorder symptoms (SEED)	Vomiting		1.22 (.61)	1-5
	Laxative use		1.09 (.44)	1-5
	Bingeing		2.26 (1.11)	1-5
BMI percentiles (categorized)	extreme underweight (<5th percentile)	17 (1.34)		
	underweight (<10th percentile)	21 (1.65)		
	healthy weight	556 (43.68)		
	overweight (>85th percentile)	234 (18.38)		
	extreme overweight (>95th percentile)	188 (14.77)		
	Missing	257 (20.19)		
Weight concerns (WCS)			66.86 (16.66)	5-100
Help-seeking attitudes (IASMHS)	Psychological openness		16.35 (4.97)	0-32
	Indifference to stigma		20.18 (5.74)	0-32
Familiarity	Familiar with a person affected by a mental illness	991 (77.85)		
	Not familiar	282 (22.15)		
Social Distance (FZSD)			.75 (.62)	0-3.43

Note. [a]Sample selection was based on a ChEDE-Q global score greater than or equal to 2.3. If not otherwise specified, sample size is n = 1,273.
